# Incidence of Atrial Fibrillation and Related Outcomes among Hospitalized Patients with Systemic Lupus Erythematosus: Analysis of United States Nationwide Inpatient Sample Database 2016–2019

**DOI:** 10.3390/jcm13061675

**Published:** 2024-03-14

**Authors:** Sushmita Mittal, Chokkalingam Siva

**Affiliations:** 1 Department of Medicine, University of Missouri, Columbia, MO 65212, USA; 2 Division of Rheumatology, University of Missouri, Columbia, MO 65212, USA; sivac@health.missouri.edu

**Keywords:** systemic lupus erythematosus, atrial fibrillation, mortality, in-hospital outcomes

## Abstract

**Background:** While patients with systemic lupus erythematosus (SLE) are known to have an increased risk of developing atrial fibrillation (AF), there is a scarcity of national population-based studies that evaluate the impact of AF on SLE. **Methods:** In this study, we use the 2016 to 2019 National Inpatient Sample (NIS) to determine the impact of AF on inpatient outcomes among adults hospitalized with systemic lupus erythematosus (SLE). Among a total of 41,004 SLE hospitalizations, 1495 (3.65%) patients had a concurrent diagnosis of AF. The baseline hospital and patient characteristics for both cohorts (SLE with AF and SLE without AF) were compared using the chi-square test and Student’s t-test while univariate and multivariate regression analysis were used to calculate the unadjusted and adjusted odds ratios (aOR) for in-hospital outcomes for both cohorts. **Results:** Our data revealed that among SLE patients, AF was associated with higher in-hospital mortality (aOR 2.07), length of stay (9.03 days), and hospital costs (USD 100,190.50) along with increased incidence of non-ST-elevation myocardial infarction (NSTEMI) (aOR 2.79), pericardial effusion (aOR 2.38), cardiac tamponade (aOR 3.33), and cardiogenic shock (aOR 8.19). **Conclusion:** Our findings suggest that patients hospitalized with SLE and underlying AF may be at risk for poor clinical outcomes.

## 1. Introduction

Systemic lupus erythematosus (SLE) is a chronic autoimmune disorder of unknown etiology. It is characterized as a highly heterogeneous disease with a wide spectrum of clinical presentations that often has a relapsing and remitting course [1]. In the United States (US), SLE is reported in about 20–150 cases per 100,000, with a higher prevalence among African American women. Recent epidemiologic studies have identified infections and cardiovascular diseases as the leading causes of death in patients hospitalized with SLE [2]. 

Atrial fibrillation (AF) is the most common supraventricular arrhythmia in the US. Studies have shown that SLE is associated with a higher risk of AF, possibly due to the chronic and systemic inflammation in SLE [3,4,5]. While it is well known that AF can increase complications in patients admitted to the hospital, there is limited literature characterizing the impact of AF on in-hospital outcomes in patients hospitalized with SLE. In this study, we utilized the Nationwide Inpatient Sample (NIS) database from 2016 to 2019 to assess the association between AF and inpatient outcomes in patients with SLE.

## 2. Materials and Methods

### 2.1. Data Source

We conducted a retrospective cohort study of adult patients with a history of SLE with or without a secondary diagnosis of AF across the US. Analysis was performed using data from the National Inpatient Sample (NIS) database from January 2016 to December 2019. The NIS is the largest all-payer publicly available database of hospitalizations in the US that was developed for the Healthcare Cost and Utilization Project (HCUP). Unweighted, it contains de-identified data on demographics, discharge diagnoses, procedures, hospital charges, comorbid conditions, and outcomes from a sample of more than 7 million hospital stays each year. The diagnoses and procedures used in this study were identified using the International Classification of Diseases, Tenth Edition, Clinical Modification/Procedure Coding System (ICD-10 CM/PCS), which was implemented on 1 October 2015, as the standardized coding system. Institutional review board approval is not required when using this dataset since all the patient information is de-identified.

### 2.2. Analytic Sample

The study population included adults aged 18 and older with a primary diagnosis of SLE, identified using the ICD-10 code of M32.x. The study population was then divided into two groups: SLE without AF (control group) and SLE with AF (study group). Our data analysis could not discriminate between patients who had a pre-existing (comorbidity) versus new-onset AF (complication) because NIS lacks “present on admission” flags that accompany its secondary diagnosis codes. Patients with missing demographic data, age < 18 years old, and a secondary diagnosis of atrial flutter were excluded from the study.

### 2.3. Variables

Variables were divided into patient level, hospital level, and illness severity:

- Patient level: Age, race, sex, comorbidities, and median income quartile based on the patient’s ZIP code
- Hospital level: Location, bed size, teaching status, and region.
- Illness severity: Mortality, length of stay (LOS), total hospital cost, non-ST-elevation myocardial infarction (NSTEMI), pericardial effusion, cardiac tamponade, cardiac arrest, and cardiogenic shock

The Elixhauser comorbidity score, which captures 31 common comorbidities, was used to estimate comorbidity burden.

### 2.4. Study Outcomes

Our primary outcome of interest was inpatient mortality between patients with AF compared to those without AF. Secondary outcomes included development of NSTEMI, pericardial effusion, cardiac tamponade, cardiac arrest, cardiogenic shock, length of stay (LOS), and total hospital cost. (ICD-10 codes provided in Table 1). 

### 2.5. Statistical Methods

Statistical analysis was performed using STATA software version 17 (StataCorp LLC, College Station, TX, USA). All data analysis was performed using weighted samples following HCUP NIS guidelines. Baseline hospital and patient characteristics for both cohorts (SLE with and without AF) were compared using the Pearson’s chi-square test for categorical variables and the Student’s t-test for continuous variables. Univariate and multivariate linear regression analysis were used to assess factors that impacted hospital costs and length of stay. Univariate and multivariate logistic regression analysis were used to assess factors that impacted mortality and rates of NSTEMI, pericardial effusion, cardiac tamponade, cardiac arrest, and cardiogenic shock. The potential confounders that were adjusted for this regression analysis included age, gender, race/ethnicity, median yearly income based on the patient’s ZIP code, the patient’s comorbidities (using Elixhauser Comorbidity Index), hospital location, hospital bed size, hospital teaching status, and geographical location. A *p* value of <0.05 was set as the threshold for statistical significance. 

## 3. Results

### 3.1. Patient and Hospital Characteristics

A total of 41,004 patients with SLE as their primary diagnosis were hospitalized between January 2016 and December 2019. Of these, 1495 (3.65%) patients had a secondary diagnosis of AF. There were 39,504 patients with SLE without a diagnosis of AF, which represented the control group of our study. The mean age of patients in the AF group was 56.22 versus 37.33 in the group without AF. Patients in both the SLE groups, with and without AF, were predominantly female. In terms of the racial distribution, almost half of the patients in the SLE and AF group were White (49.83%, *p*-value < 0.001) while most of the patients in the SLE without AF group were Black (46.68%, *p*-value < 0.001). There were no significant differences between the two groups regarding hospital bed size, hospital location, or hospital region. Further baseline characteristics of SLE patients with and without AF can be seen in Table 2. 

### 3.2. Length of Hospital Stay, Total Hospital Charges, and In-Hospital Mortality

The patients in the SLE with AF group had a mean LOS of 9.03 days, while those without AF had a mean LOS of 6.47 days. In-hospital LOS remained higher even after adjusting for age, sex, race, median household income for ZIP code, hospital region, hospital bed size, hospital location, and Elixhauser score using multivariate linear regression (adjusted odds ratio (aOR): 1.44, [95% confidence interval (CI): 0.28–2.60], *p* = 0.015). 

The mean total hospital charge was also found to be higher in the AF group (USD 100,191 vs. USD 69,604) even after multivariate linear regression analysis (adjusted mean difference (aMD): USD 19,915.93, [95% CI: (USD 946.87– USD 38,884.99], *p* = 0.040). 

All-cause unadjusted in-hospital mortality was significantly higher for patients in the AF group than in the without AF group (4.01% vs. 1.06%). The in-hospital mortality remained higher in the AF group even after multivariate logistic regression analysis after adjusting for confounders (aOR: 2.07, [95% CI: 1.08–3.94], *p* = 0.028). Further details can be seen in Table 3 and Table 4. 

### 3.3. Incidence of NSTEMI, Pericardial Effusion, Cardiac Tamponade, Cardiac Arrest, and Cardiogenic Shock

The SLE patients with AF had higher rates of NSTEMI (2.34% vs. 0.53%), pericardial effusion (14.72% vs. 7.30%), cardiac tamponade (2.34% vs. 0.77%), and cardiogenic shock (2.01% vs. 0.25%). Similar findings were obtained using multivariate logistic regression analysis, which are detailed in Table 3 and Table 4. Our data revealed that patients in the AF group did not have increased rates of cardiac arrest compared to the non-AF group (0.33% vs. 0.39%). 

**Figure 1:**
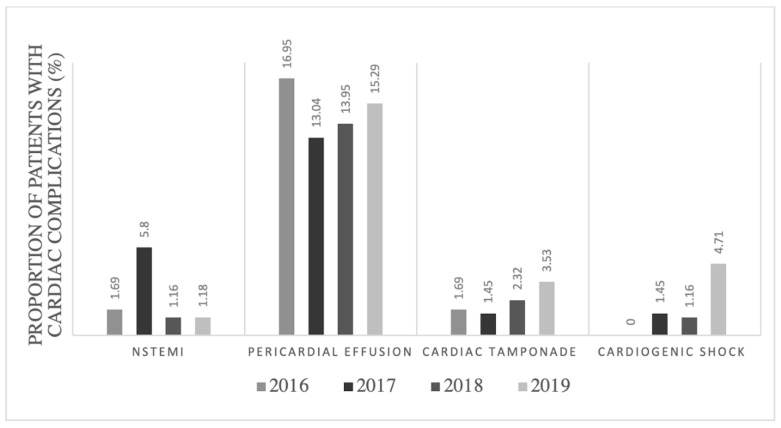
Proportion of patients in the systemic lupus erythematosus (SLE) with atrial fibrillation (AF) group with cardiac complications (%) by year. NSTEMI: non-ST-elevation myocardial infarction.

Our study also compared the yearly rates at which these outcomes occurred in patients who were in the SLE with AF group. Our data revealed that the rates of pericardial effusion, cardiac tamponade, and cardiogenic shock increased from 2016 to 2019. Meanwhile, the rate of NSTEMI occurrence was highest in 2017 (5.8%) and decreased to 1.18% in 2019. The rate of pericardial effusion increased from 2018 (13.95%) to 2019 (15.29%) and the rates of cardiac tamponade (3.53%) and cardiogenic shock (4.71%) were highest in 2019. Further details can be seen in Figure 1. 

## 4. Discussion

Our study revealed that from 2016 to 2019, 3.78% (1495/40,999) of patients with SLE had a secondary diagnosis of AF. Although studies have shown that SLE is an independent risk factor for AF development, the exact pathogenesis of this association is not clearly understood. Some studies suggest that chronic inflammation from SLE can cause structural and electrical cardiovascular remodeling that can increase the risk of AF [3]. Furthermore, SLE is associated with several inflammatory markers, including C-reactive protein (CRP), tumor necrosis factor (TNF)-a, interleukin (IL)-6, and IL-8, which have been linked to a greater risk of AF [6]. Other studies have also proposed that the increased incidence of AF could be due to the therapeutic agents used in SLE, like methylprednisolone and glucocorticoids [7]. One study conducted by Christiansen et al. revealed that glucocorticoid use increased the risk of AF by twofold [8]. Some studies suggest that this could be due to steroid-induced modifications of cardiac ion channels and shortening of the effective refractory period [9]. 

Several studies have shown that Black females are three times more likely to develop SLE than White females [10]. While our study revealed that patients in both groups were predominantly female, patients in the SLE with AF group were mostly White while those in the SLE without AF group were mostly Black. This observation is consistent with several studies that suggest that Black patients have a lower prevalence of AF compared to White patients, despite having a higher incidence of AF risk factors like hypertension, diabetes, and obesity [11,12]. This discordance has been termed a “racial paradox” and warrants further investigation of genetic, socioeconomic, and environmental determinants of health to explain these differences [13]. In terms of patient age, our data revealed that patients in the SLE with AF group were around 20 years older than the patients in the SLE without AF group (56 vs. 37 years old, respectively). While in theory, this could explain the increased incidence of worse outcomes in the SLE with AF group, our analysis found that AF leads to worse in-hospital outcomes even when we included age as a potential confounder in the multivariate regression analysis. 

There are no studies that have examined the effects of AF on in-hospital outcomes for SLE; however, there are studies that have investigated the effect of AF on other rheumatic diseases. Our study revealed that SLE patients with AF were more likely to develop pericardial effusion, cardiac tamponade, cardiogenic shock, and NSTEMI. A similar association was seen in a study performed by Basyal et al. revealed that the presence of AF in systemic sclerosis is associated with pericardial effusion (16.5%) and cardiac tamponade (19%), with higher indices compared to the overall population of patients with systemic sclerosis [14]. Another study performed by Subahi et al. revealed that in patients with acute myocarditis, AF independently increased rates of both cardiogenic shock (18.2%) and cardiac tamponade (1.9%) [15]. 

The exact pathology behind the association between AF and cardiac outcomes in SLE is uncertain. While pericardial effusion, cardiac tamponade, and cardiogenic shock are known to be potential complications of AF ablation, these complications are quite rare and likely do not explain the increased incidence seen in our study. One recent systematic review performed by Gupta et al. revealed that out of 83,236 patients who underwent AF ablation, only 1% and 0.7% experienced complications of cardiac tamponade and pericardial effusion, respectively [16]. Furthermore, our data analysis revealed that from 2016 to 2019, there were no documented cases of any SLE patients who underwent a catheter ablation, either as a primary or secondary procedure. Therefore, other explanations for the association between AF and the increased incidence of cardiac outcomes in SLE need to be examined. Other factors, like rapid ventricular rates associated with AF, can possibly worsen the underlying acute cardiac conditions in SLE, like pericarditis and myocarditis, and increase rates of cardiac complications. Our study lacks data on the heart rates associated with patients in the AF group, so the exact pathogenesis of this association is not clear and needs to be further investigated. It is also important to note that the rates of pericardial effusion, cardiac tamponade, and cardiogenic shock in the SLE with AF group have increased from 2016 to 2019, so further investigation needs to be carried out on how to decrease this incidence.

Several studies have shown that SLE significantly increases the risk of developing acute coronary syndromes (ACS) due to the high incidence of atherosclerosis compared to the general patient population. This was seen in one study performed by Asanuma et al. who found that the mean calcification score in the patients with SLE was significantly higher than those without SLE (68.9 vs. 8.8 respectively) [17]. Our study found that patients with AF were four times more likely to develop an NSTEMI compared to those without AF. This correlation between AF and NSTEMI is thought to be due to a decrease in coronary blood flow during AF. One study performed by Luo et al. found that patients with AF had poor coronary blood flow compared to those who were in sinus rhythm [18]. Interestingly, our study did not find any observed cases of STEMI in the SLE with AF group. These findings concur with a study performed by Soliman et al. which revealed that AF was associated with an increased risk of NSTEMI but not STEMI [19]. 

Our study also revealed that AF increased rates of in-hospital mortality, length of stay, and hospitalization costs in patients with SLE. One study performed by Khan et al. investigated the impact of AF on rheumatic diseases (RD) and revealed that in-hospital mortality, length of stay, and hospitalization costs were higher in the RD patients with AF group compared to those without AF [20]. Although AF is known to cause high rates of complications in general, the findings in our study are significant considering only 1495 patients out of 41,004 SLE patients had a diagnosis of AF over a 3-year period.

Our results have clinical significance as the incidence of AF continues to increase among patients with SLE. This is supported in a study performed by Lim et al. in Korea that revealed that there is an increased incidence of AF among patients with SLE compared to those without SLE (3.69 compared to 0.94 per 1000 person-years, respectively) [21]. Our data revealed that SLE patients with AF had much higher rates of NSTEMI, pericardial effusion, cardiac tamponade, in-hospital mortality, total charge, and length of stay despite only 3.65% of SLE patients having a secondary diagnosis of AF within a 3-year period. This indicates that further investigation is required to determine how the incidence of AF can be lowered in those with SLE. 

Currently, there are no guidelines on the use of electrocardiograms or echocardiographs in the early detection of cardiac manifestations of SLE. With the increasing rate of AF and AF-related complications among SLE patients, considerations can be made to implement serial cardiac screening to detect early cardiac involvement. This was performed by one prospective study by Godoy et al. which examined 48 SLE patients with serial cardiac exams (including chest X-ray, electrocardiogram, echocardiogram, cardiac stress test, and coronary angiography) every 3 years to determine their prognostic value. Results of his study revealed that among the patients with abnormalities on their initial cardiac examination, 46.2% of them demonstrated worsening at reevaluation, indicating the importance of cardiac screening and follow-up in SLE patients. This study did not reveal any findings of AF when performing serial electrocardiograms; however, this could be due to the low sample size of 48 patients along with the low likelihood of detecting paroxysmal AF on a standard 12-lead electrocardiogram [22]. Non-invasive diagnostic tools like 24 h Holter monitoring or 14-day electrocardiogram patches can be useful to detect paroxysmal AF, as described by Cheng et al. and Chua et al. [23,24]. 

### Limitation and Strengths

Although our study used a well-known database, it has several limitations. Our study heavily relied on ICD-10 codes, which are susceptible to coding errors that can affect outcomes. Furthermore, the NIS does not differentiate individual patients, so recurrent hospitalizations or transfers to other hospitals can appear as distinct observations within the study period. NIS also lacks “present on admission” flags that accompany its secondary diagnosis codes, which makes it difficult to distinguish those with a pre-existing versus new-onset diagnosis of AF. Furthermore, this makes it difficult to determine if the AF was transiently developed after common SLE complications like infection, sepsis, pericarditis, myocarditis, et cetera. The database also lacks data on medications used to treat SLE, which could potentially contribute to the negative outcomes seen in our study. Moreover, our study does not include outpatient data or long-term outcomes, so the incidence and mortality rates of the primary and secondary outcomes may be underestimated. There is also no data on the severity, prognosis, and specifics of SLE, which could act as potential confounders in our study. For example, some SLE patients could have antiphospholipid autoantibodies that are associated with prothrombotic states and atherosclerosis risk that could increase the cardiovascular complications discussed in our study [25]. It is also possible that our data underestimated the number of SLE patients with a secondary diagnosis of paroxysmal and unspecified AF since it is possible that some patients have not been officially diagnosed with these previously. Despite the limitations, our study does have strengths. Our study includes a large sample size that is representative of the general US population.

## 5. Conclusions

This study examined the impact of AF on in-hospital outcomes for patients with SLE. With the increasing incidence of AF in the US, it is vital for healthcare providers to recognize the poor outcomes that AF can have on SLE patients. Our study revealed that SLE patients with AF had higher rates of NSTEMI, pericardial effusion, cardiac tamponade, in-hospital mortality, total charge, and length of stay. These results are significant considering that only 3.65% of SLE patients had a secondary diagnosis of AF within a 3-year period. Moreover, patients in the AF group were found to have increasing rates of pericardial effusion, cardiac tamponade, and cardiogenic shock from 2016 to 2019. Cardiac disease is common among patients with SLE and having underlying arrhythmias such as AF can cause significant morbidity, mortality, and financial burden in these patients. Further research is required to assess potential interventions that can decrease the incidence of these cardiac complications among patients with SLE and AF.

## Figures and Tables

**Table 1:** International Classification of Diseases, Tenth Edition, Clinical Modification and Procedure Coding System (ICD-10 CM) Codes used in this study.

Variable	ICD-10 CM Code
Systemic Lupus Erythematosus	M32.0, M32.10, M32.11, M32.12, M32.13, M32.14, M32.15, M32.19, M32.8, M32.9
Atrial Fibrillation	I48.0, I48.11, I48.19, I48.20, I48.21, I48.91, I48.92
Non-ST-Elevation Myocardial Infarction	I21.4
Cardiac Tamponade	I31.4
Cardiogenic Shock	R57.0
Cardiac Arrest	I46.2, I46.8, I46.9

**Table 2:** Baseline characteristics of systemic lupus erythematosus hospitalizations with and without atrial fibrillation.

Patient Characteristics	SLE without AF	SLE with AF	*p*-Value
Observations	39,504	1495	
Sex (%)			
Female	86.85%	78.93%	<0.0001
Male	13.15%	21.07%	
Race (%)			
White	22.18%	49.83%	<0.0001
Black	46.68%	31.27%	
Hispanic	21.45%	11.68%	
Asian or Pacific Islander	5.07%	3.78%	
Native American	0.5%	0.34%	
Other	4.13%	3.09%	
Mean Age (years)	37.33	56.22	
Elixhauser Comorbidity Index Score (%)			
0	0%	0%	
1	7.71%	0%	
2	15.88%	1%	
3	19.50%	5.33%	
4	56.90%	93.65%	
Hospital Bed Size (%)			
Small	14.09%	17.39%	0.2258
Medium	24.90%	22.41%	
Large	61.02%	60.20%	
Hospital Location (%)			
Rural	2.81%	4.68%	0.0821
Urban non-teaching	14.35%	16.39%	
Urban teaching	82.84%	79.93%	
Hospital Region (%)			
Northeast	20.40%	23.41%	0.3570
Midwest	17.29%	18.73%	
South	42.60%	37.79%	
West	19.71%	20.07%	
Median Household Income (%)			
0–25th percentile	38.87%	32.77%	0.0011
26–50th percentile	24.27%	19.59%	
51–75th percentile	21.68%	25.34%	
>76th percentile	15.18%	22.30%	
Year			
2016	25.06%	19.73%	0.0553
2017	25.54%	23.08%	
2018	25.11%	28.76%	
2019	24.30%	28.43%	

SLE: systemic lupus erythematosus; AF: atrial fibrillation.

**Table 3:** Incidence of in-hospital outcomes in systemic lupus erythematosus patients with and without atrial fibrillation.

Clinical Outcomes	SLE without AF (*n* = 39,509)	SLE with AF (n = 1495)
NSTEMI	0.53%	2.34%
Pericardial Effusion	7.30%	14.72%
Cardiac Tamponade	0.77%	2.34%
Cardiogenic Shock	0.25%	2.01%
Cardiac Arrest	0.39%	0.33%
Inpatient Mortality	1.06%	4.01%
Mean Length of Stay	6.47	9.03
Mean Total Hospital Charge	USD 69,604.81	USD 100,190.50

SLE: systemic lupus erythematosus; AF: atrial fibrillation; NSTEMI: non-ST-elevation myocardial infarction.

**Table 4:** Unadjusted and adjusted odds ratio for the in-hospital outcomes in systemic lupus erythematosus patients with atrial fibrillation.

Clinical Outcomes	Unadjusted OR (95% CI)	Adjusted OR (95% CI)	*p*-Value for Adjusted OR
NSTEMI	4.49 (1.99–10.08)	2.79 (1.16–6.69)	0.021
Pericardial Effusion	2.19 (1.57–3.05)	2.38 (1.67–3.43)	<0.0001
Cardiac Tamponade	3.08 (1.42–6.66)	3.33 (1.46–7.56)	0.004
Cardiogenic Shock	8.07 (3.22–20.26)	8.19 (2.98–22.49)	<0.0001
Cardiac Arrest	0.85 (0.12–6.28)	0.82 (0.10–6.59)	0.851
Inpatient Mortality	3.89 (2.13–7.12)	2.07 (1.08–3.94)	0.028
Mean Length of Stay	2.57 (1.47–3.67)	1.44 (0.28–2.60)	0.015
Mean Total Hospital Charge	USD 30,655.93 (USD 13,385.17–USD 47,786.30)	USD 19,915.93 (USD 946.87–USD 38,884.99)	0.040

SLE: systemic lupus erythematosus; AF: atrial fibrillation; NSTEMI: non-ST-elevation myocardial infarction; OR: odds ratio; CI: confidence interval.

## Data Availability

The datasets used and/or analyzed during the current study are available from the corresponding author on reasonable request.
